# Computational identification of potential multi-drug combinations for reduction of microglial inflammation in Alzheimer disease

**DOI:** 10.3389/fphar.2015.00116

**Published:** 2015-06-05

**Authors:** Thomas J. Anastasio

**Affiliations:** Computational Neurobiology Laboratory, Department of Molecular and Integrative Physiology, Beckman Institute, University of Illinois at Urbana-ChampaignUrbana, IL, USA

**Keywords:** microglia, neurodegeneration, systems biology, computational biology, polypharmacology

## Abstract

Like other neurodegenerative diseases, Alzheimer Disease (AD) has a prominent inflammatory component mediated by brain microglia. Reducing microglial inflammation could potentially halt or at least slow the neurodegenerative process. A major challenge in the development of treatments targeting brain inflammation is the sheer complexity of the molecular mechanisms that determine whether microglia become inflammatory or take on a more neuroprotective phenotype. The process is highly multifactorial, raising the possibility that a multi-target/multi-drug strategy could be more effective than conventional monotherapy. This study takes a computational approach in finding combinations of approved drugs that are potentially more effective than single drugs in reducing microglial inflammation in AD. This novel approach exploits the distinct advantages of two different computer programming languages, one imperative and the other declarative. Existing programs written in both languages implement the same model of microglial behavior, and the input/output relationships of both programs agree with each other and with data on microglia over an extensive test battery. Here the imperative program is used efficiently to screen the model for the most efficacious combinations of 10 drugs, while the declarative program is used to analyze in detail the mechanisms of action of the most efficacious combinations. Of the 1024 possible drug combinations, the simulated screen identifies only 7 that are able to move simulated microglia at least 50% of the way from a neurotoxic to a neuroprotective phenotype. Subsequent analysis shows that of the 7 most efficacious combinations, 2 stand out as superior both in strength and reliability. The model offers many experimentally testable and therapeutically relevant predictions concerning effective drug combinations and their mechanisms of action.

## Introduction

Alzheimer Disease (AD) remains the leading neurological killer (www.alz.org). Currently there are no effective means to treat AD or even to slow its progression. As a neurodegenerative disease the pathological outcome of AD is death of neurons. For decades, research on AD has centered on the “amyloid hypothesis,” according to which an over-accumulation of the peptide amyloid-β (Aβ) causes neuron death (Hardy and Selkoe, 2002). New evidence continues to support a role for Aβ as a factor in AD (Hardy et al., 2014) but it also increasingly indicates that Aβ is not the only factor (Reitz, 2012; Skaper, 2012; Armstrong, 2014). Clinical, epidemiological, and laboratory evidence strongly implicates inflammation as a key component of AD pathogenesis (Griffin and Mrak, 2002; Von Bernhardi, 2007; Miklossy, 2008; Piazza and Lynch, 2009; Mandrekar-Colucci and Landreth, 2010; Johnston et al., 2011). That research suggests that Aβ, especially in the aged brain, can trigger a neurotoxic, inflammatory response and that pharmacological reduction of that response could be an effective way to treat AD.

A type of glial cell known as microglia mediates the inflammatory response in the brain (Ransohoff and Perry, 2009; Kettenmann et al., 2011). Microglia can express a number of different phenotypes in response to a wide range of stimuli including Aβ. Their physiology is extraordinarily complex. A recent computational model sought to characterize the responses to Aβ of microglia from young and old brains (Anastasio, 2014). That model provided new insights into microglial behavior and offered potential explanations for the observations that exposure to Aβ increases the production by young microglia of both pro- and anti-inflammatory mediators, and that old microglia tend to remain in a neurotoxic phenotype once they convert to it. The microglia model also identified certain cell-signaling pathways that could be instrumental in moving microglia away from a neurotoxic phenotype, but it did not explore the potential for drugs to influence those pathways.

The purpose of this paper is to use the microglia model to identify drugs, and specifically to identify drug combinations, that have the potential to reduce microglial inflammation in aged brains exposed to Aβ. As for the original microglia model, the *in silico* drug study presented here will exploit the synergistic strengths of two computer programming modalities, one imperative and the other declarative. Imperative programming, by far the more common modality, is designed for efficient computation while declarative programming is designed for computational analysis. The difference stems from the distinct nature of a statement in either modality. Basically, a statement in an imperative program is a command (e.g., add 3 and 3) but a statement in a declarative program is a declaration of a fact (e.g., 3 plus 3 can be replaced by 6). In an imperative program statements execute in the order in which they are listed, but in a declarative program a statement may execute or not. Consequently, in an imperative program statements are constrained to execute in only one order, while in a declarative program statements can execute in all possible orders.

The main benefit of a declarative programming environment is that it keeps track of the results of all of its different sequences of statement executions. This feature is what makes declarative programming inefficient compared with imperative programming, but it is also what makes the declarative modality so useful for analysis. Because a declarative program keeps track of its progress along all statement sequences it can be queried to obtain critical information, such as whether a specific result can ever occur, or whether a specific result can only occur if a different specific result occurs first, and so on. The main tools for analysis in declarative programming are known as state-space search and temporal-logic model-checking (Huth and Ryan, 2004). These tools are invaluable for the analysis of complex processes, and they are being applied increasingly to complex biological processes (e.g., Fisher and Henzinger, 2007).

Here an imperative program implementing the microglia model will be used efficiently to screen for efficacy all 1024 combinations of 10 drugs. All of the 10 have been approved for use by the US Food and Drug Administration (FDA), and all are small-molecule drugs that could be taken orally and absorbed gastrointestinally and could cross the blood-brain barrier. Each of the drugs targets a different element (or pair of elements) of the model. All of them were identified using the DrugBank database (www.drugbank.ca). As such they are all well-known and widely used. The efficacy of each drug combination will be quantified by the extent to which it moves simulated microglia from a neurotoxic to a neuroprotective phenotype. Then a declarative program implementing the same microglia model will be used to analyze the mechanisms of action of the most efficacious drug combinations. As befits the complex nature of microglia, analysis of the model will reveal that complex sets of interactions mediate the effects of the efficacious drug combinations. The analysis will demonstrate how a computational model can be used to identify potential multi-drug strategies for the manipulation of complex biological processes, and will identify specific combinations of approved, small-molecule drugs that could reduce inflammation in the AD brain.

## Methods

**Figure 1:**
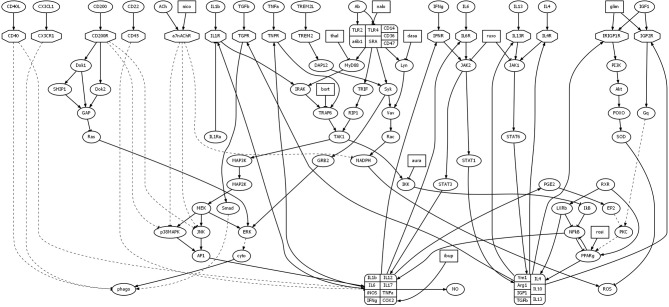
**Diagram illustrating model structure**. The model analyzed here is based on a model of microglial behavior described previously (Anastasio, 2014), to which has been added 10 drugs and their direct effects. Nodes representing drugs are rectangular, those representing receptors are octagonal, and all other nodes are elliptical. Arrows and tees represent activating and suppressing connections, respectively. Solid and dashed curves represent direct and indirect connections, respectively. For abbreviations see Table 1 or Anastasio (2014).

The goal of the study presented here was to computationally identify drug combinations with the potential to reduce microglial inflammation in AD. This study utilized a model of microglial behavior that was described in detail in a previous article (Anastasio, 2014). To this model were added 10 drugs. A schematic of the original model, along with the added drugs, is shown in Figure 1. Abbreviations of the names of the molecular species that are important for this study are listed in Table 1 (abbreviations not listed in Table 1 can be found in Anastasio, 2014). The immediate effects of each drug were set according to their main direct (proximal) effects as described in the literature, but the follow-on (distal) effects of the drugs are considered as modeling results and are described as such in Results. The first Subsection of Methods briefly summarizes the behavior of the microglia model, while the following two Subsections describe the imperative and declarative methods that were used to carry out the computational drug screens and analyses.

**Table 1:** **Abbreviations**.

**Names**	**Abbreviations**	**Elements**
Acetylcholine	ACh	ACh
Adaptor protein 1	AP1	AP1
Alzheimer Disease	AD	n/a
Amyloid-β	Aβ	Ab
Auranofin	n/a	aura
α-7 Nicotinic acetylcholine receptor	α7nAChR	a7nAChR
Bortezomib	n/a	bort
c-Jun N terminal kinase	JNK	JNK
Cluster of differentiation #	CD#	CD#
Cytochrome C oxygenase 2	COX2	COX2
Cytoskeleton	n/a	cyto
Dasatinib	n/a	dasa
E prosthanoid receptor 2	EP2	EP2
Extracellular signal-related kinase	ERK	ERK
Fractalkine	CX3CL1	CX3CL1
Glimepiride	n/a	glim
G protein q	Gq	Gq
Ibuprofen	n/a	ibup
IL1 receptor-associated kinase	IRAK	IRAK
Inhibitor of κ B kinase	IKK	IKK
Insulin-like growth factor 1	IGF1	IGF1
Insulin-like growth factor 2 receptor	IGF2R	IGF2R
Insulin receptor and insulin-like growth factor 1 receptor	IRIGF1R	IRIGF1R
Interferon γ	IFNγ	IFNg
Interleukin 1-β	IL1β	IL1b
Interleukin #	IL#	IL#
Janus kinase #	JAK#	JAK#
Lipopolysaccharide	LPS	LPS
Liver tyrosine kinase	Lyn	Lyn
Myeloid differentiation primary response protein	MyD88	MyD88
Naloxone	n/a	nalo
Nicotine	n/a	nico
Nicotinamide adenine dinucleotide phosphate (reduced)	NADPH	NADPH
Nitric oxide	NO	NO
Non-steroidal anti-inflammatory drug	NSAID	n/a
Nuclear factor κ B	NFκB	NFkB
Peroxisome proliferator-activated receptor γ	PPARγ	PPARg
Phagocytosis	n/a	phago
Prostaglandin E 2	PGE2	PGE2
Protein 38 mitogen-activated protein kinase	p38MAPK	p38MAPK
Protein kinase C	PKC	PKC
Reactive oxygen species	ROS	ROS
Receptor-interacting protein 1	RIP1	RIP1
Rosiglitazone	n/a	rosi
Ruxolitinib	n/a	ruxo
Signal transducer and activator of transcription #	STAT#	STAT#
Sma protein from *Drosophila*	Smad	Smad
Spleen tyrosine kinase	Syk	Syk
Thalidomide	n/a	thal
Toll-like receptor #	TLR#	TLR#
Toll/interleukin 1 receptor (TIR)-domain-containing adaptor-inducing interferon β	TRIF	TRIF
TNF receptor-associated factor 6	TRAF6	TRAF6
Transforming growth factor β	TGFβ	TGFb
Transforming growth factor-associated kinase 1	TAK1	TAK1
Triggering receptor expressed on myeloid cells 2 ligand	TREM2L	TREM2L
Tumor necrosis factor α	TNFα	TNFa
Vav guanine nucleotide exchange factor	Vav	Vav

*The name of each molecular species is listed along with its abbreviation and the name of the model element that represents it. Since the programming languages do not allow Greek characters they are replaced with lower-case Roman letters in model element names. To further distinguish model element names from actual molecule names they are rendered in* monotype *font. The # symbol in abbreviations or model element names stands for an arbitrary integer number. Abbreviations or model element names are not applicable (n/a) to items that are, respectively, not abbreviated in the text or do not appear in the model*.

### Microglia model behavior

The microglia model (Anastasio, 2014) represents the interactions between about 100 of the factors that are known from the literature to mediate the responses of microglia to Aβ and to other relevant stimuli. These factors are mainly molecular species (ligands, receptors, signaling molecules, transcription factors, etc.) but also include some cellular processes (e.g., phagocytosis). The microglia model is essentially a theory (albeit complex) about how microglia respond to different signals present in their environment, which is the brain parenchyma. These signals are transduced, as in other cell types, via receptors, which activate cell-signaling pathways, which effect changes in microglial functions such as upregulation or downregulation of cytokines and other mediators, activation or inactivation of phagocytosis, and so on. A key feature of microglia, as of other immune cells, is that they respond to signals that they themselves produce. These autocrine interactions constitute positive and negative feedback loops through the operation of which microglia can change their phenotype.

Microglia can assume many different phenotypes (Ransohoff and Perry, 2009; Kettenmann et al., 2011). For modeling purposes it is convenient to define different phenotypes in terms of their production of cytokines and other mediators (growth factors, toxins, enzymes, etc.) and their expression of behaviors (mainly phagocytosis). The microglia model was focused on three phenotypes that are relevant to AD pathogenesis and have been studied mainly in normal mice and in transgenic mice that overproduce Aβ (for details and references see Anastasio, 2014). These three phenotypes can be referred to as mixed, neurotoxic, and neuroprotective.

The mixed phenotype occurs in “young” microglia (from mice less than 1 year old), in which secreted or membrane-bound signals from healthy neurons, such CX3CL1 (i.e., fractalkine), are present. When young microglia are exposed to Aβ, or to other inflammatory stimuli such as LPS, they increase their production of pro-inflammatory cytokines and toxins (as exemplified by IL1β and ROS, respectively), but also increase their production of anti-inflammatory cytokines (as exemplified by IL4) and become phagocytic. The microglia model explained this by invoking the idea of an “autocrine bridge,” over which transcriptional drive could effectively “cross” from the pro- to the anti-inflammatory “side.” This occurred in the following way. Aβ (or LPS) activates the TLR complex, which initiates a signaling cascade that activates NFκB, which in turn upregulates a set of pro-inflammatory cytokines including IFNγ. IFNγ then activates the IFNR in an autocrine fashion, which activates JAK2/STAT1, which in turn upregulates a set of anti-inflammatory cytokines and other mediators including TGFβ. TGFβ then activates Smad, which in turn contributes to the activation of phagocytosis. This mixed, pro-and-anti-inflammatory phenotype is on the whole beneficial because phagocytic microglia clear Aβ, but its beneficial aspects do not occur unless the pro-to-anti-inflammatory bridge is crossed. The pro-to-anti-inflammatory bridge can be crossed by IFNγ or IL6, or potentially by other cytokines that signal via JAK2/STAT1, but removal of STAT1 severely dysregulates this mechanism.

The neurotoxic phenotype occurs in “old” microglia (from mice greater than 1 year old), in which signals from healthy neurons are absent. When old microglia are exposed to Aβ together with other inflammatory stimuli such as LPS, they increase their production of pro-inflammatory cytokines but decrease their production of anti-inflammatory cytokines and become non-phagocytic (Piazza and Lynch, 2009). The microglia model explained this by hypothesizing that a mutually antagonizing interaction between NFκB and PPARγ, which occurs in macrophages, also occurs in microglia. With this mutually antagonizing mechanism in place, the loss of healthy neuronal signals in old microglia combined with exposure to both Aβ and LPS pushed NFκB up higher than in the mixed phenotype, and that in turn pushed PPARγ down to the point where it caused downregulation of anti-inflammatory mediators. The downregulated factors included TGFβ, which was not then high enough to promote phagocytosis. The neurotoxic phenotype is damaging to neurons because of high levels of pro-inflammatory cytokines and ROS, low levels of anti-inflammatory cytokines and other factors that support neuronal maintenance, and lack of Aβ clearance through microglial phagocytosis.

A key aspect of the mixed phenotype is that many but not all anti-inflammatory mediators are upregulated. Notable among those not upregulated is IGF1 (Butovsky et al., 2005). In the model this occurs because STAT1 upregulates most anti-inflammatory mediators but downregulates IGF1. A central feature of the model is autocrine interaction, through which each pro-inflammatory cytokine can upregulate itself and other pro-inflammatory cytokines. Because the cytokines upregulated by pro-inflammatory cytokines include IFNγ (and IL6), a pro-inflammatory cytokine can even upregulate certain anti-inflammatory factors. In contrast, an anti-inflammatory factor cannot, in the model, upregulate pro-inflammatory cytokines. While an anti-inflammatory factor can upregulate itself and most other anti-inflammatory factors, it cannot upregulate IGF1 when STAT1 has been activated. Otherwise, an anti-inflammatory factor such as IL4 (or IL13) can temporarily upregulate IGF1 via STAT6 but, in the model, IGF1 must then activate its own signaling pathway via Gq in order to maintain a high level. Failure to do so leaves IGF1 vulnerable to STAT1.

Unlike the other anti-inflammatory mediators, IGF1 can downregulate pro-inflammatory cytokines. It accomplishes this essentially by reversing the transcriptional pattern that underlies the neurotoxic phenotype in the model. Specifically, IGF1 can drive up PPARγ, which then drives down NFκB, and this causes downregulation of pro-inflammatory cytokines along with upregulation of anti-inflammatory mediators. However, this beneficial influence of IGF1 is tenuous because IGF1 cannot depend on other anti-inflammatory mediators but must activate its own signaling pathway in order to maintain a high level. According to the model, the neurotoxic phenotype is persistent because microglia have only one mechanism available to escape it, but that mechanism requires sustained IGF1 expression, and that cannot occur through synergism with other pathways but depends on a single pathway that is initiated by IGF1 itself signaling though IGFR and Gq. In the model, movement away from the neurotoxic phenotype depends entirely on activation this pathway.

It is possible experimentally to reduce a pro-inflammatory microglial response, to restore Aβ phagocytosis, and to increase IGF1 expression using initially high levels of anti-inflammatory cytokines such as IL4, IL10, and IL13 (Butovsky et al., 2005; Koenigsknecht-Talboo and Landreth, 2005). In the model this phenotype can be produced though initial increase in IGF1 itself. It can also be produced through initial increase in IL4, or other anti-inflammatory cytokines that signal via JAK1/STAT6, but only if IGF1 subsequently maintains its own high level by activating Gq. In the model this phenotype is characterized by low levels of pro-inflammatory cytokines, high levels of anti-inflammatory cytokines (including IGF1), and phagocytosis of Aβ. This phenotype can be reached even from the neurotoxic phenotype provided that IGF1 activates its pathway through Gq. This microglial phenotype is considered neuroprotective because it would not damage neurons with high levels of pro-inflammatory cytokines or ROS, and it would clear Aβ and other debris through phagocytosis while it supports neural repair through secretion of growth factors and other proteins such as enzymes mediating cytoskeletal remodeling (see also Discussion).

In the model, the phenotype reached from the neurotoxic phenotype due to activation of IGF1/IGFR/Gq has ROS reduced from the high to the baseline level. For the purposes of the computational drug-combination screen presented here, the fully neuroprotective phenotype will have low ROS in addition to low pro-inflammatory cytokines, high anti-inflammatory mediators including IGF1, and a high level of Aβ phagocytosis. The goal of the computational screen is to identify which combinations of 10 approved drugs have the greatest potential to move microglia from the neurotoxic to the fully neruoprotective phenotype, and then to analyze the mechanisms of action of the most efficacious combinations. This is accomplished using data-driven computational models implemented in two different programming environments.

### Data-driven modeling framework

The original model, on which this computational drug-combination screen is based, is a model of the signaling system by which microglia adjust their phenotype according to changes in the brain's microenvironment. The microglia system model is entirely data driven in that all of the interactions represented in it are based directly on the results of experiments as reported in the primary literature. These results were obtained using molecular and cellular biology techniques (gel electrophoresis, immunohistochemistry, phagocytosis assay, etc.) that provide relative measures of expression and/or activity and are useful for making statistical comparisons, but do not provide accurate measures of quantities such as concentrations or reaction rates. For the purposes of modeling it is therefore appropriate to quantize available data into a set of discrete integer levels. In establishing the framework for the microglia system model, the data were used to determine both the structure of the system and its overall input/output behavior (also called the model truth table; see below).

Model structure was determined from studies of microglia signaling pathways. For example, using a combination of immunological receptor-blocking, gel electrophoreses, immunohistochemistry, and *in vitro* binding assay, fibrillar Aβ was shown to activate a microglial receptor complex that includes TLR2, TLR4, and CD14, leading to phosphorylation of the tyrosine kinases Lyn and Syk, which in turn phosphorylate Vav (Combs et al., 1999; Reed-Geaghan et al., 2009). The structure of the original microglia model was built up using these and many similar results. To the original model were added proximal drug effects. For example, using a combination of immunoprecipitation and gel electrophoreses, the drug dasatinib was shown to block phosphorylation of Lyn (Nam et al., 2007).

The overall input/output behavior of the microglia system was determined from studies of the responses of microglia to the stimuli Aβ or LPS, or to high levels of various mediators (e.g., IFNγ). For example, using superoxide and *in vitro* phagocytosis assays, microglia from young mice were shown to respond to fibrillar Aβ by increasing both ROS production and phagocytosis (Wilkinson et al., 2006). These and many other similar findings were used to compose a system “truth table,” which showed the effects of manipulating the levels of certain elements, designated as input elements, on the levels of certain other elements designated as output or endpoint elements. The input elements included Aβ and LPS while the output or endpoint elements included IL1β, IL4, IGF1, ROS, and phagocytosis. For the truth table, inputs such as Aβ and LPS were treated as binary (absent or present, 0 or 1), while naturally occurring molecules (e.g., IL1β or IL4) were assigned the integer levels of 5, 3, or 7 for baseline, low, or high, respectively, where low and high indicate observed, statistically significant increases or decreases from baseline, respectively. The truth table represented the findings with which the computational model had to agree.

Model structure dictated which model elements could influence with other elements and the polarity of that influence (activation or suppression), but these constraints left open many options for computationally describing how each element responded to its inputs from the other elements. The parsimonious approach taken in model creation started by setting the absolute strengths of all connections to 1 (+1 for activating connections, −1 for suppressing connections). Then the functions that determine the expression/activation level (level function) for each element were set so that overall model behavior agreed with the truth table. This trail-and-error process involved complex interactions more so that simpler interactions. Thus, elements that receive input from 1 other element take the same level as their single input element. Elements that receive input from 2 or 3 other elements either take the maximal input or take sums and/or differences between the inputs (differences, like all other levels in the model, are bound from below at 0). Only about 15% of the elements in the original microglia model received more than three inputs. Their level functions were constructed in terms of specific input patterns. The more complicated level functions could depend on the values of inputs relative to each other or to preset threshold values, and could also involve saturation values that bound the level from above. An effort was made to keep the level functions as simple as possible while forging agreement between the model and the data-derived truth table (for further details see Anastasio, 2014).

The names of the model elements correspond to the usual abbreviations for the molecules they represent (with a few corresponding to cellular processes). Model element levels are constrained to take integer values in the range [0, 10]. To distinguish model elements from actual molecules (or cellular processes) their names are written in monotype font (see Table 1). The name of the original model of the behavior of microglia in the AD brain is ADMICRO. The name of this model augmented to include the effects of 10 drugs is ADMICRODRUGS. These computational models are instantiated as computer programs. Like ADMICRO, there are two versions of ADMICRODRUGS, one written in an imperative language and the other written in a declarative language.

### Imperative programming and simulation

The imperative programming language used in this computational study is MATLAB™. MATLAB is designed for highly efficient computations, especially those that can be organized as matrix computations (the name “MATLAB” is short for “matrix laboratory”). MATLAB was used here to instantiate the ADMICRODRUGS model, to run repeated simulations of it, to perform the computational drug-combination screen, and to sort the results of the screen in order to remove redundancies.

In the MATLAB version all model elements are represented as integer variables, and all level functions assign integers in the range [0, 10] to their associated variable. For example, IL1b equals 7 when its level function has assigned it that level. To facilitate comparisons, the structure of the MATLAB program was made more similar to the Maude program (see next Subsection) by making all of its level functions conditional and placing them all within a while loop. The condition for any level function is satisfied if its execution would change the value of its associated variable, in which case the variable is updated and an update flag is set. The while loop continues as long as the update flag is set. What this means is that whenever a variable is updated, all level functions must be checked again to determine whether any of their variables could now also change value. The while loop terminates when no further updates can be made. The MATLAB version did terminate for all of the input configurations represented in the truth table.

An ancillary MATLAB program automatically ran simulations of the MATLAB version of ADMICRODRUGS starting from each input configuration, collected the terminal state endpoint values, and computed the error between model and observed endpoints over the entire truth table. This ancillary program was used efficiently to evaluate trial-and-error changes in level functions during model creation (see previous Subsection). A terminal state of critical importance for this study is the neurotoxic state (i.e., phenotype; see Subsection Microglia Model Behavior). This state is reached in old microglia from the input configuration that includes both Ab and LPS (i.e., the neurotoxic input configuration). That input configuration is the one from which the drug-combination screen began. Another ancillary MATLAB program first constructed the matrix of all possible combinations of the 10 drugs screened in this study, ran a simulation for each combination starting from the neurotoxic input configuration to find the terminal state endpoint values for that combination, computed the percent efficacy of each combination, and then removed redundancies. The percent efficacy is a measure of the closeness of an endpoint pattern to the neuroprotective pattern, expressed as a percentage (see Results). A redundancy is a specific combination of drugs that is not more effective than a combination of the same drugs minus one or more of them. Determination of drug-combination efficacies and removal of redundancies was facilitated using matrix manipulations implemented in MATLAB.

### Declarative programming and analysis

The declarative programming language used here is Maude (Clavel et al., 2007). Maude is designed to represent (i.e., model) and analyze complicated systems. A Maude model of a system is considered to be a module (the name “Maude” is derived from the word “module”). A module has an underlying algebra, which is composed of sorts (i.e., things) and operators (i.e., things done to things). The declarations in a Maude program are then based on this underlying algebra. Maude was also used here to instantiate the ADMICRODRUGS model and to analyze the model using state-space search and temporal-logic model checking.

In the Maude version of the model all elements are represented as operators that assign an integer (i.e., a sort) in the range [0, 10] to the level of that element. For example, IL1b(7) is an operator that assigns the integer 7 to the level of IL1b. Each declaration in the Maude program specifies the level function for an element in terms of the operators representing its inputs. In Maude there are two types of declarations: equations and rules. Applicable equations must execute but applicable rules may execute or not. This allows Maude to execute applicable rules in all possible orders thereby constructing the tree of all possible system states reachable given the rules (each branch is a different trajectory). Maude can search the tree for states of interest (i.e., state-space search) or can determine temporal relationships between states (i.e., temporal-logic analysis). In ADMICRODRUGS, as in ADMICRO, the level functions that mediate the autocrine interactions (i.e., those that determine the levels of the receptors for the cytokines and other mediators that the microglia themselves produce) are expressed as rules, while all other interactions are expressed as equations. Thus, Maude determines all of the system states reachable through autocrine interactions in the model.

In the Maude version, as in the MATLAB version, all level functions are conditional, but unlike the MATLAB version the conditions are checked in arbitrary order in the Maude version (this is due to the essential difference between imperative and declarative programs). Like the MATLAB version the Maude version terminates for all input configurations but, unlike the MATLAB version, the Maude version reaches more than one terminal endpoint state for some input configurations. In all cases, for all input configurations and for all drug combinations tested, one Maude terminal endpoint state matched the single MATLAB terminal endpoint state. This signified that the two versions could implement the same computation and ensured that the results were not corrupted by programming error. In addition to providing a crosscheck, the Maude version was also used to analyze the mechanisms by with the efficacious drug combinations reduced neurotoxicity in the model.

## Results

The results focus on the effects in the model of administration of 10 drugs alone and in all possible combinations. These drugs are, in alphabetical order, auranofin, bortezomib, dasatinib, glimepiride, ibuprofen, naloxone, nicotine, rosiglitazone, ruxolitinib, and thalidomide. All of these drugs are small-molecule and FDA approved (see also www.drugbank.ca). As small-molecules they can potentially cross the blood-brain barrier and exert their effects on microglia. Eight of the ten drugs normally are administered orally. The two exceptions are bortezomib and naloxone. Bortezomib is currently administered intravenously but oral forms of bortezomib are currently in clinical trial (Lawasut et al., 2012). Naloxone can be administered by various routes including orally, although the bioavailability of orally administered naloxone is low (Smith et al., 2012).

Thalidomide is a known teratogen but it has been re-approved by the FDA for limited use (Matthews and McCoy, 2003). Thalidomide reduces the level of MyD88 both by downregulating MyD88 mRNA expression and by increasing degradation of MyD88 by the proteasome (Noman et al., 2009). Bortezomib inhibits the proteasome as a main effect, but it also induces proteasome-independent degradation of TRAF6 (Fang et al., 2012). Thus, bortezomib and thalidomide both reduce inflammation by reducing signaling over inflammatory pathways. When used in conjunction, as they sometimes are clinically (Wang et al., 2014), bortezomib would lessen but not eliminate the anti-inflammatory effects of thalidomide by inhibiting the proteasome.

Naltrexone and naloxone, which block opioid receptors, and an isomer of naloxone that does not block opioid receptors, all act as antagonists of TLR4 (Hutchinson et al., 2008). The benefit of using the non-opioid isomer of naloxone is that it can block TLR4 and reduce inflammation but not block the analgesic effects of opioids. While the opioid and the non-opioid naloxones both can inhibit TLR4 signaling, the non-opioid naloxone does not also inhibit TLR2 signaling (Lewis et al., 2012). Because of its reduced side effects the model incorporates the non-opioid, rather than the opioid, isomer of naloxone. Both TLR2 and TLR4 signal via Lyn and Syk. Dasatinib blocks activation (i.e., phosphorylation) of sarcoma kinases including Lyn (Nam et al., 2007); dasatinib does not block Syk because it is not a sarcoma kinase (McDonald et al., 1997; Combs et al., 1999).

Nicotine inhibits Aβ-induced microglial ROS production by preventing the activation of NADPH (Moon et al., 2008). This ROS suppression by nicotine was prevented by blockers of the α7nAChR. Ibuprofen, a well-known NSAID and COX2 inhibitor, can reduce inflammation, microglial activation, and AD deposits in AD-transgenic mice but its efficacy is age dependent (Lim et al., 2000; Yan et al., 2003; Sung et al., 2004). Rosiglitazone is a dose-dependent agonist of PPARγ that has known anti-inflammatory properties (Loane et al., 2009). Auranofin inhibits IKK (Jeon et al., 2000, 2003), while ruxolitinib inhibits both JAK1 and JAK2 (Verstovsek, 2013). Glimepiride is an insulin secretagogue, which causes insulin release from the pancreas (Campbell, 1998). Glimepiride is known to have anti-inflammatory properties (Ingham et al., 2014). All of these drugs directly target specific model elements.

The drugs are evaluated in terms of their effects on model behavior when it is started from the old initial condition. In this case, ACh, CD22, CD200, CX3CL1, and TREM2L, which represent secreted and/or membrane-bound substances produced by young, healthy neurons, are all absent (level 0), and the inflammatory stimuli Ab and LPS are both present (level 1). The effects are quantified in terms of the changes they make in the levels of a set of five key elements designed as endpoint elements. The endpoint elements are IL1b, ROS, phago, IL4, and IGF1, which represent, respectively, pro-inflammatory cytokines, toxins, phagocytosis, anti-inflammatory cytokines, and an anti-inflammatory factor of singular importance in the model (see also Anastasio, 2014). In all model terminal states the endpoint elements take one of three levels, which are 5, 3, and 7, corresponding to base, low, and high. In the absence of any intervention, the old initial state, with Ab and LPS both present, leads to the neurotoxic terminal state (i.e., phenotype) in which pro-inflammatory cytokines are high, ROS is high, anti-inflammatory factors are low, and phago is low. This can be expressed succinctly using Maude operators: in the old case with Ab(1) and LPS(1) the terminal state has IL1b(7), ROS(7), phago(3), IL4(3), and IGF1(3).

The neurotoxic endpoint pattern and many other reference endpoint patterns are listed in Table 2. The two most important are the neurotoxic pattern (Table 2, Row 6) and the neuroprotective pattern (Table 2, Row 7), because the efficacy of any drug combination was judged by its ability to move the microglia from the neurotoxic to the neuroprotective phenotype. Expressed as vectors, the neurotoxic and neuroprotective endpoint levels are [7 7 3 3 3] and [3 3 7 7 7], respectively. Then the efficacy of any single drug or drug combination was quantified by how much it moved the endpoint vector (as moving a needle on a dial) from the neurotoxic to the neuroprotective vector, and this amount was expressed as a percentage (0% meant no movement or fully neurotoxic, while 100% meant maximal movement or fully neuroprotective). The efficacies of all single drugs and of all non-redundant drug combinations are listed in Table 3.

**Table 2:** **Model endpoints from specific start conditions with no drugs or with single drugs**.

**Row**	**Condition**	**IL1b**	**ROS**	**phago**	**IL4**	**IGF1**
1	Young microglia, AB(0), LPS(0), and no drugs (baseline)	5	5	5	5	5
2	Young microglia, AB(1), LPS(0), and no drugs (mixed)	7	7	7	7	3
3	Young microglia, AB(0), LPS(1), and no drugs (mixed)	7	7	7	7	3
4	Young microglia, AB(0), LPS(0), TNFaini(8), and no drugs	7	5	7	7	3
5	Old microglia, AB(0), LPS(0), and no drugs	7	5	3	7	3
6	Old microglia, AB(1), LPS(1), and no drugs (neurotoxic)	7	7	3	3	3
7	Old microglia, AB(1), LPS(1), but effects reversed (neuroprotective)	3	3	7	7	7
8	Young microglia, AB(0), LPS(1), and thal(1)	5	7	5	5	5
9	Young microglia, AB(0), LPS(1), and bort(1)	5	7	5	5	5
10	Young microglia, AB(1), LPS(0), and nico(1)	5	5	5	5	5
11	Young microglia, AB(0), LPS(1), and nico(1)	5	5	5	5	5
12	Young microglia, AB(1), LPS(0), and ibup(1)	5	7	7	7	5
13	Young microglia, AB(0), LPS(1), and rosi(1)	5	7	7	7	5
14	old microglia, AB(0), LPS(0), and rosi(1)	5	5	7	7	5
15	Young microglia, AB(0), LPS(1), and aura(1)	5	7	5	5	5
16	Young microglia, AB(0), LPS(0), TNFaini(8), and ruxo(1)	7	5	5	5	5
17	Young microglia, AB(0), LPS(1), and glim(1)	5	5	7	7	5

*The first seven rows list a set of reference phenotypes with no drugs (including mixed, neurotoxic, and neuroprotective). The following 10 rows list specific phenotypic modifications due to certain drugs administered singly. The abbreviation TNFaini stands for the initial level of TNFa*.

**Table 3:** **Model endpoints starting from the old initial condition with Ab(1) and LPS(1) and with drugs either singly or in specific combinations**.

**Row**	**aura**	**bort**	**dasa**	**glim**	**ibup**	**nalo**	**nico**	**rosi**	**ruxo**	**thal**	**IL1b**	**ROS**	**phago**	**IL4**	**IGF1**	**% effect**
1	1										7	7	7	7	3	26
2		1									7	7	3	3	3	0
3			1								7	7	3	3	3	0
4				1							7	5	3	3	3	5
5					1						7	7	3	3	3	0
6						1					7	7	3	7	3	17
7							1				7	5	3	3	3	5
8								1			7	7	3	3	3	0
9									1		7	7	3	3	5	5
10										1	7	7	3	3	3	0
11	1								1		7	7	5	5	5	10
12						1			1		7	7	5	5	5	10
13	1					1					5	7	3	5	5	17
14	1			1		1					5	5	7	7	5	51
15				1				1			5	5	7	7	5	51
16				1			1	1			5	3	7	7	5	72
17	1			1			1				5	3	7	7	5	72
18				1		1	1				5	3	7	7	5	72
19				1	1						3	5	7	7	7	78
20				1	1		1				3	3	7	7	7	100

*The neurotoxic endpoint pattern, which proceeds from the old initial condition with Ab(1) and LPS(1) and with no intervention, is shown in Table 2, Row 6*.

### The effects of each drug administered alone in the model

In ADMICRODRUGS, each drug affects one or more targets, which are the elements of the original ADMICRO. Each drug alters the level of its direct target(s) in a manner that persists into the terminal state of the model. The follow-on effect of each drug also alters the levels of other elements, such as signaling-pathway and transcription-factor elements. Some single drugs may also alter the levels of the endpoint elements IL1b, ROS, phago, IL4, and IGF1. Note that the effects of the drugs on their direct targets are explicitly set according to experimental findings (see previous Subsection for references), but all follow-on effects can be considered as modeling results.

Thalidomide reduces the level of MyD88. It does that in part by increasing degradation of MyD88 by the proteasome, so it would be less effective in conjunction with bortezomib, the main effect of which is to inhibit the proteasome. In ADMICRODRUGS, thal is set to reduce the level of MyD88 by 2 if bort is absent but only by 1 if bort is present. By reducing the level of MyD88 in the model, thal impairs the MyD88-initiated signaling that results in activation of transcription factor NFkB, but only in young microglia. For example, thal by itself is effective in young microglia challenged by LPS. In the young case with Ab(0) but LPS(1) and without any intervention, the terminal state has MyD88(1), with pathway elements IRAK(7), TRAF6(7), TRIF(1), RIP1(1), and TAK1(7), and transcription factors NFkB(7) and PPARg(5). The endpoint states are IL1b(7), ROS(7), phago(7), IL4(7), and IGF1(3) (Table 2, Row 3). With NFkB(7) and PPARg(5), the pro- and anti-inflammatory mediators (except for IGF1) are expressed at the high level, and ROS is high but phago is also high. If this initial state includes thal(1), then the terminal state has MyD88(0), IRAK(4), TRAF6(5), TRIF(1), RIP1(1), TAK1(5), NFkB(5), and PPARg(5), and the endpoint states are IL1b(5), ROS(7), phago(5), IL4(5), and IGF1(5) (Table 2, Row 8). With NFkB(5) and PPARg(5), the pro-inflammatory cytokines, including IFNg, are held at base, and this prevents the microglial system from crossing the IFNg autocrine bridge that causes upregulation of anti-inflammatory mediators (except for IGF1; note that IFNg autocrine-bridge crossing causes IGF1 downregulation in the model). Consequently phago is also held at base, although ROS still rises to high. Thus, in the young case with LPS(1), initial thal(1), by suppressing MyD88, prevents LPS from activating NFkB, which prevents upregulation of pro-inflammatory cytokines, and this is consistent with observation (Noman et al., 2009).

Although thal is effective by itself in young microglia challenged by LPS, its effectiveness is quite different in old microglia challenged by Ab and LPS, which leads to the neurotoxic state in the model. Among the non-endpoint elements, the unaltered neurotoxic state has MyD88(2), IRAK(8), TRAF6(8), TRIF(2), RIP1(2), TAK1(9), NFkB(9), and PPARg(3), and the endpoint states are IL1b(7), ROS(7), phago(3), IL4(3), and IGF1(3) (Table 2, Row 6). If the initial state includes thal(1), then the terminal state has MyD88(0), IRAK(6), TRAF6(7), TRIF(2), RIP1(2), TAK1(9), NFkB(9), and PPARg(3), and the endpoint states are IL1b(7), ROS(7), phago(3), IL4(3), and IGF1(3) (Table 3, Row 10). In the model, thal reduces MyD88 to 0, and this slightly reduces the levels of IRAK to 6 and TRAF6 to 7. However, TRIF provides a pathway through RIP1 to TAK1 that parallels MyD88, and TRIF and RIP1 are unaltered by thal. Combined with RIP1 at 2, TRAF6 at 7 is still enough to drive NFkB to 9 and that pushes PPARg down to 3. With NFkB(9) and PPARg(3), the pro- and anti-inflammatory mediators all assume their unaltered neurotoxic levels. Adding to its ineffectiveness by itself, thal(1) does not reduce ROS or enhance phago either. Thus, thal by itself is ineffective in old microglia in altering any of the endpoints from their neurotoxic levels.

Because high-dose bortezomib is cytotoxic, ADMICRODRUGS simulates a relatively low dose. In the model, bort is set to hold the level of TRAF6 to base (rather than push TRAF6 to low) under all circumstances in which TRAF6 would exceed base, thereby reducing TRAF6 activation. The effects of bort by itself are similar to those of thal. By itself, bort does not alter any of the neurotoxic levels of the endpoint elements in old microglia challenged by Ab and LPS in the model (Table 3, Row 2). In contrast, in young microglia challenged by LPS alone, bort holds all pro- and anti-inflammatory mediators (including IGF1) to base and also holds phago at base although ROS still rises to high (Table 2, Row 9). Thus, bort reduces the response of young microglia to LPS alone, but is ineffective by itself in combating the neurotoxic state induced in old microglia by Ab and LPS in the model.

To simulate the effects of nicotine, nico is set to activate a7nAChR to level 2, and this causes indirect inhibition of NADPH, JNK, and p38MAPK. In the absence of any intervention, young microglia with initial Ab(1) or initial LPS(1) reach a terminal state that has a7nAChR(1), NADPH(2), ROS(7), JNK(7), p38MAPK(7), AP1(7), NFkB(7), and TNFa(7). If either initial state has nico(1) then the terminal state has a7nAChR(2), NADPH(1), ROS(5), JNK(5), p38MAPK(5), AP1(5), NFkB(5), and TNFa(5). Here nico raises a7nAChR to 2, which pushes NADPH down to level 1, and that pushes ROS down to the base level of 5. It also holds JNK and p38MAPK to 5. This keeps AP1 at 5, at which level it cannot increase NFkB. This keeps all pro-inflammatory cytokines including TNFa and IFNg at the base level (of 5), and that prevents the autocrine-bridge crossing that would otherwise elevate anti-inflammatory factors (except IGF1) and in turn elevate phago (Table 2, Rows 1–3, 10 and 11). In that nico reduces activation of NADPH, JNK, and p38MAPK and blocks production of ROS and TNFa in young microglia challenged by Ab or LPS, the model is consistent with observation (Shytle et al., 2004; De Simone et al., 2005; Moon et al., 2008). The neurotoxic state has a7nAChR(0), NADPH(2), ROS(7), JNK(10), p38MAPK(10), AP1(10), NFkB(9), and TNFa(7). If the initial state includes nico(1), then the terminal state, as in the previous case, has a7nAChR(2), which pushes NADPH down to level 1, which pushes ROS down to level 5. Also, the initial nico(1) again holds JNK, p38MAPK, and AP1 to the base level of 5, but that does not result in reduction of pro-inflammatory cytokines or augmentation of anti-inflammatory factors or phago because the level of NFkB is still at the over level of 9. Thus, nico reduces ROS from high to base but does not otherwise alter the neurotoxic phenotype by itself in the model (Table 3, Row 7).

To simulate the non-opioid isomer of naloxone, nalo is set to block activation of TLR4 by Ab or LPS, but not to block activation of TLR2 by Ab. (Note that LPS specifically activates TLR4; Necela et al., 2008; Park et al., 2009.) Among the non-endpoint elements, the unaltered neurotoxic state has TLR2(1), TLR4(2), MyD88(2), IRAK(8), TRAF6(8), NFkB(9), and PPARg(3). If the initial state includes nalo(1), then the terminal state has TLR2(1), TLR4(0), MyD88(1), IRAK(7), TRAF6(7), NFkB(7), and PPARg(5). TLR2 is not sensitive to LPS but still responds to Ab, which drives TLR2 to level 1 and then TLR2 drives MyD88 to level 1. This level of MyD88 is enough to drive IRAK, TRAF6, and NFkB to the high level of 7, which is enough to keep the pro-inflammatory cytokines high, but not enough to drive PPARg low, so the IFNg autocrine bridge can be crossed. This drives up most of the anti-inflammatory mediators except IGF1. The result is IL1b(7), IL4(7), and IGF1(3) (Table 3, Row 6). The initial nalo(1) does not alter ROS, which stays high, nor does it alter phago, which stays low. Thus, nalo by itself increases some anti-inflammatory factors but not IGF1, nor does it decrease ROS, or decrease pro-inflammatory cytokines, or increase phago in the neurotoxic phenotype of the model.

To simulate the effects of dasatinib, dasa is set to block activation of Lyn by TLR2 or TLR4, but to have no effect on Syk. In the absence of any intervention, the neurotoxic state of old microglial in the model has Lyn(2), Syk(2), NADPH(2), and ROS(7). If the initial state includes dasa(1) then the terminal state has Lyn(0), Syk(2), NADPH(2), and ROS(7). Both Lyn and Syk drive Vav. In the model, Syk at level 2 is enough to bring Vav and then NADPH to level 2 and that is enough to drive ROS to high even though Lyn is blocked by dasa. Thus, dasa by itself does not reduce ROS nor does it alter the levels of any of the other endpoint elements in the neurotoxic state of the model (Table 3, Row 3).

The effects of ibuprofen are represented in ADMICRODRUGS according to its well-known main mechanism: ibup is set to prevent the increase in PGE2 due to COX2. The follow-on effects of ibup depend on age and inflammatory stimulants in a complex way. In the young case without intervention and with Ab(1) but LPS(0), the terminal endpoint states are IL1b(7), ROS(7), phago(7), IL4(7), and IGF1(3), and the terminal levels of some of the non-endpoint elements are PGE2(7), EP2(7), PKC(0), PPARg(5), and NFkB(7). When this young initial state includes ibup(1) these terminal states are IL1b(5), ROS(7), phago(7), IL4(7), IGF1(5), PGE2(0), EP2(0), PKC(5), PPARg(8), and NFkB(4) (Table 2, Rows 2 and 12). By inhibiting COX2, ibup blocks PGE2 and subsequent EP2 production, and the resulting disinhibition of PKC increases PPARg, which decreases NFkB. The result is a decrease in pro-inflammatory cytokines to the base level, retention of high levels of most anti-inflammatory cytokines and with an increase in IGF1 to base, and no decrease in ROS but also no decrease in phago. However, in the old case with both Ab(1) and LPS(1), which leads to the neurotoxic terminal state, NFkB is 9, PPARg is 3, and ibup has no effect on model endpoints (Table 3, Row 5). If the neurotoxic state of the model does indeed reflect the microglial state in the aged and AD brain, as conjectured here (see Discussion), then these model attributes are consistent with experimental findings. They show that NSAIDs including ibuprofen are effective in reducing inflammation and (through phagocytosis) Aβ plaque formation in AD-transgenic mice, but only if chronic NSAID treatment is begun while the animals are still young (Lim et al., 2000; Yan et al., 2003; Sung et al., 2004).

To simulate rosiglitazone, rosi is set to strongly (but not fully) activate PPARg. If the young initial state has LPS(1) but Ab(0) and there is no intervention, then the terminal endpoint are IL1b(7), ROS(7), phago(7), IL4(7), and IGF1(3), and the terminal level of PPARg is 5 and of NO is 7. If the initial state includes rosi(1), which raises PPARg to 8, then NO falls to 5 and the terminal state has IL1b(5), ROS(7), phago(7), IL4(7), and IGF1(5) (Table 2, Rows 3 and 13). Thus, rosi brings the pro-inflammatory cytokines down to base and IGF1 up to base, while phago and the other anti-inflammatory factors retain the high level. Although ROS is not reduced, NO is reduced. In the old case without inflammatory stimuli (i.e., LPS and Ab are both 0), the untreated terminal state has IL1b(7), ROS(5), phago(3), IL4(7), and IGF1(3) (Table 2, Row 5). Additionally PPARg is 5 and NO is 7. Including rosi in this case, which again raises PPARg to 8, again brings the pro-inflammatory cytokines down to base and IGF1 up to base, and the other anti-inflammatory factors retain the high level. Additionally phago is increased to 7 while NO is decreased to 5 (Table 2, Row 14). In that rosi reduces IL1b and NO in the young case with LPS(1) and in the old case without any inflammatory stimulus, the model is consistent with experimental findings (Loane et al., 2009). Because the synergistic combination of Ab and LPS in the old case raises NFkB to 9, which pushes PPARg down to 3 despite rosi, rosi has no effect by itself on the neurotoxic state of the model (Table 3, Row 8).

To simulate auranofin, aura is set to hold IKK to 4 if TAK1 is less than over but to 5 if TAK1 reaches the over level. In both cases aura has some efficacy. Again, if the young initial state has LPS(1) but Ab(0) and with no intervention, then the terminal endpoints are IL1b(7), ROS(7), phago(7), IL4(7), and IGF1(3), and among non-endpoints IKK and NFkB are both 7. If the initial state includes aura(1) then IKK and NFkB are both held at 4 and the terminal state has IL1b(5), ROS(7), phago(5), IL4(5), and IGF1(5) (Table 2, Rows 3 and 15). Thus, aura blocks the response of young microglia to LPS in the model, except that it does not reduce ROS. In that aura reduces the LPS-induced increases in IKK and NFkB in the model it is consistent with experimental findings (Jeon et al., 2000, 2003). Because aura acts directly on IKK, which activates NFkB, aura does have an effect by itself in reducing the severity of the neurotoxic state. Again, if the old initial state has Ab(1) and LPS(1) and there is no intervention then the neurotoxic terminal state occurs. It has endpoints IL1b(7), ROS(7), phago(3), IL4(3), and IGF1(3), and the non-endpoints IKK and NFkB are both 9. If that initial state has aura(1) then IKK and NFkB are held at 5 and the endpoints are IL1b(7), ROS(7), phago(7), IL4(7), and IGF1(3) (Table 3, Row 1). Thus aura by itself brings the model from the neurotoxic phenotype, which results in old microglia with Ab(1) and LPS(1), to the mixed phenotype that results in young microglia with Ab(1) or LPS(1) but not both (Table 2, Rows 2 and 3).

To simulate ruxolitinib, ruxo is set to hold both JAK1 and JAK2 to the base level. In the young case with enhanced initial TNFa (TNFaini(8)) but with Ab and LPS both 0 and no other intervention, the model terminal state has endpoints IL1b(7), ROS(5), phago(7), IL4(7), and IGF1(3). It also has IL6(7) and TNFa(7), and JAK1, JAK2, and STAT1 are all 7. If this initial state also includes ruxo(1) then JAK1, JAK2, and STAT1 are reduced to 5, and the terminal state has endpoints IL1b(7), ROS(5), phago(5), IL4(5), and IGF1(5), and also IL6(7) and TNFa(7) (Table 2, Rows 4 and 16). This is consistent with findings that ruxolitinib inhibits TNFα-induced JAK1/2 and STAT1 activation but does not significantly reduce TNFα-induced production of IL1β, IL6, and TNFα itself (Yarilina et al., 2012; Verstovsek, 2013). Although ruxo does not reduce pro-inflammatory cytokines in this case it shifts all anti-inflammatory factors to the base level, and this represents a reduction for all the anti-inflammatory factors except IGF1, which is actually augmented from low to base by ruxo. This last effect alone is recapitulated in the neurotoxic state, in which ruxo by itself does not alter the levels of any endpoint elements except for IGF1, which takes the base rather than the low level (Table 3, Row 9).

To simulate glimepiride, glim is set to raise the activation of IRIGF1R to level 7 and of IGF2R to level 6, to reflect findings that IGF2R is less sensitive to insulin than is the hybrid insulin-IGF1 receptor IRIGF1R (Fernandez and Torres-Aleman, 2012). Again, if the young initial state has LPS(1) but Ab(0) and there is no intervention, then the terminal endpoint states are IL1b(7), ROS(7), phago(7), IL4(7), and IGF1(3), and additionally TNFa(7). If this initial state includes glim(1) then the terminal endpoints are IL1b(5), ROS(5), phago(7), IL4(7), and IGF1(5), and also TNFa(5) (Table 2, Rows 3 and 17). Thus glim decreases the levels of the pro-inflammatory endpoints and ROS from high to base but does not decrease the high level attained by phago and the anti-inflammatory factors except for IGF1, which is actually increased from low to base. In that glim opposes the LPS-induced increases in pro-inflammatory cytokines including IL1b and TNFa, the model is consistent with experimental observations in microglia (Ingham et al., 2014). Because glim fully activates IRIGF1R it also reduces ROS from high to base in the neurotoxic phenotype but, because glim does not fully activate IGF2R, it does not alter any other neurotoxic endpoint states by itself in the model (Table 3, Row 4).

### Temporal-logic analysis of antagonistic drug combinations

Half of the drugs applied by themselves reduced the severity of the neurotoxic phenotype, with efficacies ranging from 5 to 26% (Table 3, Rows 1–10). The two most effective single drugs were aura (26% efficacy) and nalo (17% efficacy). There were three pairs of drugs that were actually less effective than one member of the pair alone. The drug ruxo reduced the efficacies of aura and of nalo (Table 3, Rows 11 and 12). Through its inhibition of both JAK1 and JAK2, ruxo disrupts the autocrine interactions by which pro-inflammatory cytokines can drive up most anti-inflammatory mediators but drive down IGF1. The result is that ruxo prevents aura or nalo from driving up IL4 and some other anti-inflammatory mediators, and in the case of aura it also prevents the increase in phago that is due in part to the increase in TGFb. However, ruxo prevents the neurotoxic decrease of IGF1 that aura or nalo alone do not prevent but that ruxo does prevent by itself (Table 3, Row 9).

On its own, aura moves the neurotoxic phenotype in a neuroprotective direction by raising anti-inflammatory cytokines like IL4 to high and also raising phago to high. With aura alone, temporal logic shows that NFkB never rises above base but AP1 is always above high, and that leads to high expression of pro-inflammatory cytokines like IL1b and IFNg. Also STAT1 eventually reaches high, which implies that IL4 and TGFb are high but IGF1 is low. In this way aura allows crossing of the autocrine bridge by which pro-inflammatory cytokines like IFNg raise the levels of anti-inflammatory factors like IL4 and TGFb but lower the level of IGF1. The analysis also shows that with aura alone, ERK can still reach a level above high and this allows TGFb to drive phago to high in this case. With aura and ruxo together, STAT1 does not eventually reach high because ruxo suppresses JAK2, so the rise in most anti-inflammatory mediators along with the fall in IGF1 does not occur, but NFkB still never rises above base, so all the anti-inflammatory mediators (including IGF1) and phago stay at base.

The situation with nalo is similar. With nalo alone, NFkB does rise above base but never rises above high, so NFkB drives up the pro-inflammatory cytokines but NFkB never reaches the over level necessary to override the autocrine bridge. Therefore, as with aura, the autocrine bridge is crossed and STAT1 eventually reaches high with nalo also, and drives up most anti-inflammatory cytokines including IL4 and TGFb while it drives down IGF1. However, nalo does not allow ERK to rise above high, which it would need to do in this case to allow TGFb to drive up phago. With nalo and ruxo together, STAT1 does not eventually reach high because ruxo suppresses JAK2, so the rise in the anti-inflammatory mediators such as IL4 and TGFb, and the fall in IGF1, does not occur. Still, NFkB never rises above high, so the anti-inflammatory mediators as well as phago stay at base. Thus, ruxo thwarts both aura and nalo by suppressing JAK2 (the suppression by ruxo of JAK1 is less relevant here).

The third antagonistic drug pair is aura and nalo. Their interference can be explained by their respective effects on ERK and NFkB. In the signaling pathway leading from Ab and LPS stimulation, nalo works further upstream than aura by blocking TLR4. In consequence, nalo suppresses ERK but aura does not. In combination, nalo reduces the percent efficacy of aura alone to the level of nalo alone, but the endpoint pattern of the aura and nalo combination is different from the pattern due to nalo alone (Table 3, Rows 1, 6, and 13). With aura and nalo together, NFkB is suppressed further than with either drug alone and never even reaches the base level. Having NFkB below base implies that pro-inflammatory cytokines like IL1b and IFNg are at kept at base, which prevents autocrine-bridge crossing and so further implies that anti-inflammatory factors like IL4, TGFb, and IGF1 are kept at base. With aura and nalo together, unlike aura alone but as with nalo alone, the rise in ERK is suppressed and that causes phago to fall to low in this case. In all of the cases examined in this Subsection, which are aura alone, nalo alone, each separately combined with ruxo, or aura and nalo together without ruxo, ROS is not suppressed but reaches the high level. Although nalo antagonizes aura, and aura undoes some of the benefit of nalo, the aura and nalo combination is synergistic when glim is added to the mix (see next Subsection).

### Temporal logic analysis of synergistic drug combinations

There were 376 combinations with percent efficacies of 50% or greater, but the overwhelming majority of these were redundant in the sense that one or more drugs in the combination did not increase the overall efficacy of the combination. Removing all redundancies reduced the number of combinations with efficacies of 50% or greater to only seven (Table 3, Rows 14 to 20). Of all ten drugs, glim was the only drug present in all seven of the synergistically efficacious combinations. Temporal-logic analysis shows that glim by itself keeps ROS to the base level. While glim also helped keep ROS at or below base in all seven synergistic combinations, its contribution to efficacy was not limited to ROS reduction.

The drug glim greatly improves the efficacy (to 51%) of the aura and nalo combination in one of the two stable states associated with this combination (Table 3, Row 14). In addition to reducing ROS, glim allows PPARg to rise to the over level along the trajectory leading to the more efficacious state. This raises the level of anti-inflammatory factors such as IL4 and TGFb to high, and this rise is self-sustaining in the stable state in which STAT6 attains the high level. The effects of nalo on ERK allow TGFb to drive up phago. Due to the combination of aura and glim, NFkB is kept below base so the pro-inflammatory cytokines cannot rise above base and cannot drive IGF1 below base.

There are three stable states associated with the combination of glim and rosi. The most efficacious of these has the same percent efficacy (of 51%) and endpoint pattern as the aura, glim, and nalo combination (Table 3, Rows 14 and 15), and the efficacious states of aura/glim/nalo and of glim/rosi are similar but occur via different mechanisms. Along its most efficacious trajectory, the combination glim/rosi works mainly by driving PPARg above high, which pushes NFkB below base. With these key transcriptions factors at those levels, the pro-inflammatory cytokines are held at base and cannot drive down IGF1, which also stays at base, but the other anti-inflammatory factors are driven high. The glim/rosi combination does not affect the TLR2/TLR4 signaling pathway so ERK is allowed to rise and, in this case, that allows TGFb to drive phago to the high level. Adding nico to the glim/rosi combination increases its efficacy to 72% (Table 3, Rows 15 and 16). The main contribution of nico to the glim/nico/rosi combination is to further reduce ROS by always keeping the level of NADPH less than 2.

Two other combinations have the same efficacy (of 72%) and the same terminal endpoint pattern as the glim/nico/rosi combination. These are the aura/glim/nico and the nalo/glim/nico combinations (Table 3, Rows 17 and 18). Two stable states are associated with aura/glim/nico. For both of them, due to aura and glim, NFkB never rises above base. In consequence, pro-inflammatory factors such as IL1b, and the anti-inflammatory IGF1, eventually settle at base. Due to the combined influences of glim and nico, ROS is held at low in both stable states. Along the more efficacious trajectory, an increase in PPARg above high drives STAT6 to the high level, and that leads to sustained high expression of anti-inflammatory factors such as IL4 and TGFb. Because TLR2/TLR4 signaling is not disrupted by the aura/glim/nico combination, ERK eventually rises above high, and that allows TGFb to drive phago to the high level in this case.

There are also two stable states associated with the nalo/glim/nico combination, and the mechanism by which the more efficacious state occurs is similar to that of the aura/glim/nico combination with two small differences. First, because nalo blocks TLR4 signaling, ERK is held below high, but that also allows TGFb to drive phago to high in this case. Second, PPARg eventually reaches base in both combinations, but that in conjunction with STAT1 at base is enough to keep IGF1 at base for the nalo/glim/nico combination. Otherwise, the mechanisms for keeping the pro-inflammatory cytokines at base, the anti-inflammatory factors (except IGF1) at high, and ROS at low are similar between these two combinations (Table 3, Rows 17 and 18).

The most efficacious combinations are glim/ibup and glim/ibup/nico (Table 3, Rows 19 and 20), and their mechanisms are the simplest. Both combinations are associated with only one stable state, as are each of glim, ibup, and nico separately. With glim by itself, PPARg rises to the over level but it does not stay there, while with ibup by itself PPARg eventually falls to low. However, with glim and ibup in combination, PPARg rises to over and stays there due to the rise in Gq by glim and the fall in EP2 by ibup. By raising Gq to high this combination ensures sustained high expression of IGF1, which drives up the level of PPARg. When PPARg reaches the over level it drives NFkB low, and this configuration of these key transcription factors brings forth the expression pattern characteristic of the neuroprotective state in which all pro-inflammatory cytokines are low and all anti-inflammatory mediators, including IGF1, are high. With glim/ibup, ERK can still rise above high and that allows TGFb to drive phago to high in this case, and glim reduces ROS to base. All that separates this endpoint pattern from the fully neuroprotective pattern is that ROS is reduced all the way to low in the latter.

The glim/ibup combination just described has 78% efficacy (Table 3, Row 19). Adding nico to the mix raises its efficacy to 100% (Table 3, Row 20). The combination glim/ibup/nico exhibits all the same mechanisms as the glim/ibup combination but it also keep ROS at low due to the activation by nico of the a7nAChR. Thus, in the model, microglia can be brought from the neurotoxic state to the fully neuroprotective state by a combination of glim, ibup, and nico.

## Discussion

The key finding of this study is the identification of seven specific combinations of FDA-approved, small-molecule drugs that could potentially reduce microglial inflammation in the aged and AD brain (Table 3, Rows 14–20). Many lines of evidence indicate that the basal state of microglia becomes more inflammatory with age (for review see Wong, 2013). This chronic neuroinflammation is associated with higher levels of pro-inflammatory cytokines, and possibly also with higher levels of certain anti-inflammatory cytokines. The microglia model takes on this phenotype in the old case in the absence of Ab and LPS (Table 2, Row 5) due to the lack of ACh, CD22, CD200, CX3CL1, and TREM2L. These neuronal factors are associated with young, healthy neurons and they suppress microglial inflammation in the model. This is consistent with experimental findings (for references see Anastasio, 2014) with the possible exception of CX3CL1, findings for which are ambiguous. Some studies indicate that CX3CL1, also known as fractalkine, can partially suppress the pro-inflammatory response of microglia (Cardona et al., 2006), while other studies indicate that it can suppress phagocytosis of Aβ and partially enhance the pro-inflammatory response of microglia in transgenic AD mice (Lee et al., 2010). A recent study in transgenic AD rats reported increased CX3CL1 along with increased IL1β, TNFα, and COX2 even in young animals (Hanzel et al., 2014). The authors of that study proposed that release of CX3CL1 or other factors by Aβ-damaged neurons could exacerbate or even possibly trigger the inflammatory response in the AD brain.

While it seems obvious that age-related and Aβ-related damage to neurons would contribute to a neurodegenerative process, age is not the sole cause of AD and neither is Aβ since Aβ accumulation in brain is frequently observed in aged individuals who show little or no dementia (Lee et al., 2004; Castellani et al., 2009). It is likely that factors in addition to age and Aβ can push the brain into a neurodegenerative state. In this study, that factor is LPS, which is meant to represent the kind of debris the builds up from a lifetime of fighting bacterial infections. Such debris can persist as an inflammatory stimulus and invade the aged and AD brain (Miklossy, 2008; Bibi et al., 2014).

Experimental evidence indicates that young and old microglia exposed to Aβ alone (i.e., without LPS) take on similar phenotypes except for phagocytosis. Specifically, both young and old microglia exposed to Aβ have increased production of ROS and of pro- and anti-inflammatory mediators (except for decreased production of IGF1), but young microglia have increased phagocytosis while old microglia have decreased phagocytosis (for references see Anastasio, 2014). When old microglia are exposed to Aβ and LPS together *in vitro* they also have increased production of ROS and of pro-inflammatory cytokines and decreased phagocytosis, but with Aβ and LPS together old microglia have *decreased* production of anti-inflammatory mediators (including IGF1) (Piazza and Lynch, 2009). Deprived of whatever protection the anti-inflammatory mediators may offer (see below), this phenotype is considered neurotoxic. It has not been described *in vivo* but is here conjectured to occur in aged and AD brains, due to the possible infiltration of inflammatory stimuli besides Aβ (such as LPS) that can also activate the TLR complex. The computational drug-combination screen presented here took this neurotoxic phenotype, as initiated by a combination of Aβ and LPS in old microglia, as a starting point, but only some of the findings are dependent on LPS.

The neurotoxic phenotype simulated here is triggered in the old case by Ab and LPS together, signaling through TLR4, but is produced and maintained by a transcription-factor pattern in which NFkB is expressed at its highest level while PPARg is expressed at its lowest level. All seven of the synergistically efficacious drug combinations work by reversing that pattern, in part or in whole. The seven efficacious combinations involve six of the ten drugs: aura, glim, ibup, nalo, nico, and rosi. Of those, only nalo works by blocking activation of TLR4 by LPS (and Ab). All of the others (except nico) work on elements that more directly determine the levels of NFkB and PPARg (see Results). If, as conjectured here, a neurotoxic phenotype occurs in the aged and AD brain because of co-stimulation by Aβ and other TLR4 ligands (such as LPS), then the model suggests that drug combinations that include a TLR4 blocker, such as the non-opioid form of naloxone, would help to reduce that neurotoxicity (opioid forms that block both TLR2 and TLR4 might help even more). If, on the other hand, AD does not involve co-stimulation between Aβ and other TLR ligands, then the neurotoxic phenotype described here can be considered as a worst-case scenario of microglial activation. In that case the model suggests that certain combinations of auranofin, glimepiride, ibuprofen, and rosiglitazone would help to reduce microglial inflammation in AD. It further suggests that nicotine can contribute by reducing ROS in some of those combinations.

The two most efficacious combinations were glim/ibup and glim/ibup/nico. The combined effects of glim and ibup were enough to completely reverse the neurotoxic transcriptional pattern involving NFkB and PPARg, resulting in low expression of pro-inflammatory cytokines, high expression of anti-inflammatory mediators including IGF1, and high phago. Reduction of ROS from base to low by the addition of nico completed the conversion from the fully neurotoxic to the fully neuroprotective phenotype. Among the anti-inflammatory factors expressed at a high level in the neuroprotective phenotype is TGFb (see Results). Various mechanisms of neuroprotection by TGFβ through direct action on neurons have been described (for review see Caraci et al., 2012). It is possible that certain anti-inflammatory mediators including IL4 and IGF1 may also be neuroprotective via mechanisms that go beyond suppression of the neurotoxic microglial state.

Two further aspects of the highly efficacious glim/ibup and glim/ibup/nico combinations stand out. First, only combinations including both glim and ibup were able completely to reverse the neurotoxic NFkB-PPARg pattern. Second, only combinations including both glim and ibup were associated with only one stable state. These two aspects are related.

The neuroprotective transcriptional pattern (NFkB low but PPARg high) can be maintained only when IGF1 is able to sustain its own high expression though activation of the Gq pathway. This mechanism is tenuous because it cannot be maintained through synergism with other anti-inflammatory mediators but can only be sustained by IGF1 itself (see Subsection Microglia Model Behavior). The efficacious drug combinations that did not include both glim and ibup could not completely reverse the neurotoxic NFkB-PPARg pattern, nor could they even guarantee, for all orders of updates of the autocrine receptors (i.e., all state trajectories), that the partial reversal they could produce would be maintained. Thus, the glim/ibup and the glim/ibup/nico combinations emerge from the analysis as the most efficacious as well as the most reliable. The model suggests that the combination of glimepiride and ibuprofen could effectively and reliably reduce microglial inflammation in AD, and could also reduce ROS with the addition of nicotine.

The impetus for the computational drug-combination screen described here is similar to that which drives other computational and experimental approaches in the emerging area of polypharmacology (Keith et al., 2005; Hopkins, 2008; Lehar et al., 2009; Xie et al., 2012). What sets this computational study apart is its use of both imperative and declarative programming modalities to simulate and analyze a pathological system. This dual approach allows both identification of potentially effective drug combinations and detailed analysis of their putative mechanisms of action. As such, the model generates predictions of both therapeutic and basic-science relevance.

The most direct way to test the specific predictions generated here would be to use old microglia exposed to both Aβ and LPS *in vitro* (for *in vitro* experiments, insulin itself would be used in place of glimepiride). Validation *in vitro* that the efficacious drug combinations identified here actually do reduce microglial inflammation would provide strong justification for *in vivo* tests, which could lead to clinical trials. However, failure to validate model predictions would also be useful, and in that case the ability to use declarative analysis tools is critical. While descriptions of the mechanisms of action of the various drug combinations may seem tedious they are nevertheless essential, because if the predictions are not validated, then experiments involving the receptors, signaling molecules, transcription factors, and other elements can show us specifically where the model goes wrong and how to correct it. Yoking computational and experimental efforts would spur development of a model of ever increasing explanatory power, and one that would produce increasingly valuable predictions concerning multi-drug therapies aimed at the highly complex and severely damaging inflammatory component of neurodegenerative diseases including AD.

### Conflict of interest statement

The author declares that the research was conducted in the absence of any commercial or financial relationships that could be construed as a potential conflict of interest.
